# Podoplanin expression in the development and progression of laryngeal squamous cell carcinomas

**DOI:** 10.1186/1476-4598-9-48

**Published:** 2010-03-02

**Authors:** Juan P Rodrigo, Dario García-Carracedo, María V González, Gonzalo Mancebo, Manuel F Fresno, Juana García-Pedrero

**Affiliations:** 1 Department of Otolaryngology-Head and Neck Surgery, Hospital Universitario Central de Asturias, Instituto Universitario de Oncología del Principado de Asturias, Universidad de Oviedo, Oviedo, Asturias, Spain; 2 Department of Pathology, Hospital Universitario Central de Asturias, Instituto Universitario de Oncología del Principado de Asturias, Universidad de Oviedo, Oviedo, Asturias, Spain

## Abstract

**Background:**

Podoplanin expression is attracting interest as a marker for cancer diagnosis and prognosis. We therefore investigated the expression pattern and clinical significance of podoplanin during the development and progression of laryngeal carcinomas.

**Results:**

Podoplanin expression was determined by immunohistochemistry in paraffin-embedded tissue specimens from 84 patients with laryngeal premalignancies and 53 patients with laryngeal squamous cell carcinomas. We found podoplanin expression extending from the basal to the suprabasal layer of the epithelium in 37 (44%) of 84 dysplastic lesions, whereas normal epithelium showed negligible expression. Patients carrying podoplanin-positive lesions had a higher laryngeal cancer incidence than those with negative expression reaching borderline statistical significance (51% *versus *30%, *P *= 0.071). Podoplanin expression in laryngeal carcinomas exhibited two distinct patterns. 20 (38%) cases showed diffuse expression in most tumour cells and 33 (62%) focal expression at the proliferating periphery of tumour nests. High podoplanin expression was inversely correlated with T classification (*P *= 0.033), disease stage (*P *= 0.006), and pathological grade (*P *= 0.04). There was a trend, although not significant, towards reduced disease-specific survival for patients with low podoplanin levels (*P *= 0.31) and diffuse expression pattern (*P *= 0.08).

**Conclusions:**

Podoplanin expression increases in the early stages of laryngeal tumourigenesis and it seems to be associated with a higher laryngeal cancer risk. Podoplanin expression in laryngeal squamous cell carcinomas, however, diminishes during tumour progression. Taken together, these data support a role for podoplanin expression in the initiation but not in the progression of laryngeal cancers.

## Background

Head and neck squamous cell carcinoma (HNSCC) is the fifth most common cancer worldwide. Although in most studies HNSCC is considered as a single type of carcinoma, important differences in the clinical and biological behaviour have been observed depending on the location of the tumours (i. e. oral cavity, oropharynx, hypopharynx and larynx). Patients with HNSCC have benefited greatly from the latest advances in surgical techniques, radiation therapy and chemotherapy. However, despite the advancements in local control and overall quality-of-life achieved with the use of combined modality therapies, the survival rates for HNSCC have not improved significantly over the past two decades [1]. Hence, novel methods of cancer detection and prognostication need to be developed. Recent advances in genomic and basic research have increased our understanding of the molecular processes governing tumour formation and progression. HNSCC is a heterogeneous disease involving dysregulation of multiple pathways linked to cellular differentiation, cell cycle control, apoptosis, angiogenesis, and metastasis [2]. Thus, much work is focused on the identification of better biologic and molecular factors that may serve as prognostic and predictive markers [3].

Human podoplanin is a 38-kDa mucin-type transmembrane glycoprotein consisting of 162 amino acids. In normal tissues, podoplanin is expressed in kidney podocytes [4], skeletal muscle, placenta, lung and heart [5], in myofibroblasts of the breast and salivary glands, in osteoblasts and mesothelial cells [6]. Occasionally, focal expression of podoplanin can be found in circumscribed areas of the basal layer of the human epidermis [7]. As podoplanin is expressed on lymphatic but not on blood vessel endothelium, it has been widely used as a specific marker for lymphatic endothelial cells and lymphangiogenesis in many species [4], including HNSCC [8]

The expression of podoplanin is up-regulated in a number of different human cancers, including squamous cell carcinoma of the oral cavity, the lung, the cervix, the oesophagus, and the skin, in dysgerminomas of the ovary and granulosa cell tumours, in mesothelioma, and in many tumours of the central nervous system (CNS) [5,7,9-12]). In addition, recent experimental results have demonstrated that podoplanin mediates a pathway leading to collective cell migration and invasion *in vivo *and *in vitro *[12].

The expression of podoplanin in human cancers and its relationship with tumour invasion raises the possibility that podoplanin expression could be used as a biomarker for diagnosis and prognosis. Supporting this notion, podoplanin has been identified as a marker of malignant transformation and poor prognosis in oral cancer [13,14]. Since HNSCC may behave differently depending on the tumour site, in this work we analysed podoplanin expression in a series of squamous cell carcinomas and premalignant lesions of the larynx to ascertain the role of podoplanin in both malignant transformation and tumour progression and its clinical significance in laryngeal cancer.

## Methods

### Patients and Tissue Specimens

Surgical tissue specimens from 84 patients with premalignant lesions of the larynx and 53 patients with laryngeal squamous cell carcinomas who underwent surgical treatment at the Hospital Universitario Central de Asturias between 1996 and 2004 were retrospectively collected, following institutional review board guidelines. Informed consent was obtained from each patient. Representative tissue sections were obtained from archival, paraffin-embedded blocks and the histological diagnosis was confirmed by an experienced pathologist (M.F.F.). The sections were selected for study as follows: In premalignant lesions, the entire lesion was included in one block and therefore the section used for histological diagnosis was subsequently stained and evaluated. In carcinomas, one representative section from the middle of the tumour that also included the tumour border (containing interfaces between the tumour nests and stroma) and normal adjacent epithelium was selected for staining.

Premalignant lesions were classified into the categories of mild, moderate or severe dysplasia following the WHO classification [15]. Fourteen (17%) lesions were classified as mild dysplasia, 26 (31%) as moderate dysplasia, and 44 (52%) as severe dysplasia/carcinoma "in situ" (CIS). All patients were men, with a mean age of 64 years (range 36-83 years). All of them were smokers, and 43 were also habitual alcohol drinkers. The patients with a diagnosis of premalignant lesion and cancer within the next six months were excluded from the study. All patients were treated by excisional biopsy using stripping microflap excision with cold instruments. A complete macroscopic exeresis of the lesion was performed in all cases, but the microscopic margins were not addressed. Patients were followed-up for a minimum of 60 months or until progression to malignancy occurs.

All patients with laryngeal squamous cell carcinoma included in this study were surgically treated. All had a single primary tumour, microscopically clear surgical margins and received no treatment prior to surgery. A total of 14 (26%) patients received post-operative radiotherapy (this was administered to stage IV patients). All but one of the patients were male, the mean age was 63 years (range 33 to 86 years). All of them were smokers and 45 were also habitual alcohol drinkers. The characteristics of the patients studied and the clinico-pathological features of their tumours are shown in Table 1. The stage of the disease was determined after the surgical resection of the tumour according to the Tumor, Lymph Node, Metastases TNM system of the International Union Against Cancer (6th edition). The histological grade was determined according to the degree of differentiation of the tumour (Broders' classification).

Patients were followed up for at least 36 months.

**Table 1:** Clinico-pathological characteristics of the laryngeal squamous cell carcinomas and correlations with podoplanin expression.

Parameter	No (%)	Low podoplanin expression (%)	High podoplanin expression (%)	*P*
Tumour site				
- Supraglottis	23 (43)	19 (83)	4 (17)	0.01
- Glottis	30 (57)	14 (47)	16 (53)	
pT classification				
- T1	14 (26)	5 (36)	9 (64)	0.033
- T2	13 (25)	7 (54)	6 (46)	
- T3	17 (32)	13 (76)	4 (24)	
- T4	9 (17)	8 (89)	1 (11)	
pN classification				
- N0	38 (72)	23 (61)	15 (39)	0.53
- N1-3	15 (28)	10 (67)	5 (33)	
Disease stage				
- I	16 (30)	5 (31)	11 (69)	0.006
- II	9 (17)	5 (56)	4 (44)	
- III	10 (19)	7 (70)	3 (30)	
- IV	18 (34)	16 (89)	2 (11)	
Degree of differentiation				
- Well differentiated	32 (60)	16 (50)	16 (50)	0.04
- Moderately differentiated	16 (30)	13 (81)	3 (19)	
- Poorly differentiated	5 (10)	4 (80)	1 (20)	
Recurrence				
- No recurrence	42 (79)	25 (60)	17 (40)	0.42
- Loco-regional recurrence	7 (13)	4 (57)	3 (43)	
- Distant metastasis	4 (8)	4 (100)	0 (0)	

### Immunohistochemistry

The formalin-fixed, paraffin-embedded tissues were cut into 4-μm sections and dried on capillary-gap glass slides (DakoCytomation). The sections were deparaffinized with standard xylene and hydrated through graded alcohols into water. Antigen retrieval was performed by heating 5 minutes in a pressure cooker with citrate buffer (pH 6.0). Tissue slides were incubated overnight at 4°C in a humid chamber with mouse IgG anti-podoplanin monoclonal antibody (clone D2-40, Covance Inc. formerly Signet Catalog No. 730-01) at 1:100 dilution and staining was done by using the DakoCytomation Envision Plus peroxidase mouse system and diaminobenzidine chromogen as substrate (DakoCytomation).

Counterstaining with haematoxylin for 1 minute was the final step. After staining, the slides were dehydrated through graded alcohols and mounted with a cover slip using a standard medium. Expression of podoplanin in lymphatic endothelial cells within the stroma served as an internal positive control. Negative controls with an omission of the antiserum from the primary incubation were also included. The slides were analysed randomly by three of the authors, blinded to clinical data.

To allow for a comparison between the results from this study and those previously reported [13,14], two different scoring systems were used to evaluate podoplanin expression in laryngeal premalignant lesions and laryngeal carcinomas. In premalignant lesions, immunostaining was scored using a similar scoring system to that described by Kawaguchi *et al *[14], as follows: (0) if no expression was observed in any part of the epithelium, (1) expression restricted to the basal layer of the epithelium, (2) expression in the basal and suprabasal layers at one area, and (3) suprabasal layer expression at two or more areas.

In laryngeal carcinomas, podoplanin expression was scored as described by Yuan *et al*: [13]: quantity scores from 0 to 5 were respectively assigned if 0%, 1% to 10%, 11% to 30%, 31% to 50%, 51% to 80%, and 81% to 100% of the tumour cells were positive. The staining intensity was rated on a scale of 0 to 3 (0 = negative, 1 = weak, 2 = moderate, and 3 = strong). The raw data were then converted to a German Immunoreactive Score (IRS) by multiplying the quantity and staining intensity scores. Theoretically, the scores could range from 0 to 15. An IRS score above the median (7 or higher) was considered high reactivity and 0 to 6 weak. The consensus opinions were used to assign final IRS scores to the disputed cases before data analysis.

### Statistical analyses

All statistical analyses were performed using the SPSS statistical software version 8.0 (SPSS Inc., Chicago, IL., USA). The χ^2 ^test or Fisher's exact test were used for comparison between categorical variables and Student's t-test for parametric continuous variables. Time-to-event analyses were calculated using the Kaplan-Meier product-limit estimate. Differences between times were analysed by the log-rank method. Multivariate Cox proportional hazards models were used to examine the relative impact of either variables demonstrated to be statistically significant in univariate analysis or those variables likely to have an effect on outcome. All tests were two-sided. *P *values of ≤ 0.05 were considered statistically significant.

## Results

### Podoplanin expression during laryngeal tumourigenesis

**Figure 1:**
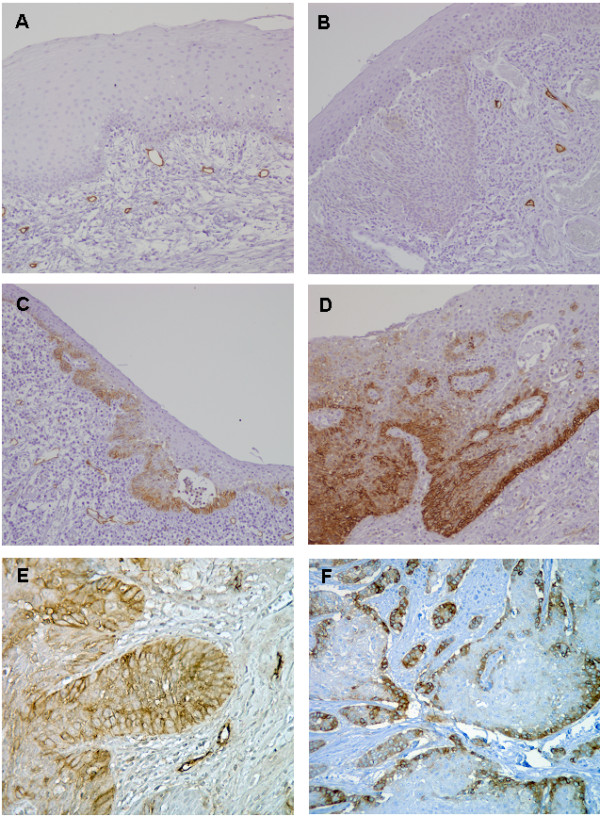
**Immunohistochemical analysis of podoplanin expression**. Representative examples of podoplanin expression in normal laryngeal epithelium (A), dysplastic epithelium with negative podoplanin staining (B), dysplastic epithelium with positive podoplanin staining scored as 2 (C) and 3 (D), laryngeal carcinomas showing diffuse podoplanin expression (E) and focal expression (F).

Podoplanin expression was consistently detected with high immunoreactivity in endothelial cells of lymphatic vessels, as expected according to its established role as lymphatic marker. In contrast, podoplanin expression in normal laryngeal epithelium was negligible or restricted to small clusters of cells within the basal layer that showed mainly membranous and cytoplasmic staining (Fig. 1A). Podoplanin expression in dysplastic laryngeal epithelium was highly variable, from no expression (Fig. 1B) to high expression (Fig. 1C), yielding a predominant membranous pattern at the basal layer. In some cases, the expression extended to suprabasal layer or above at one or multiple areas (Fig. 1C-1D).

Among the 84 dysplastic lesions analysed, 10 cases (12%) showed no detectable podoplanin expression in the epithelium (scored as 0), 37 (44%) showed podoplanin expression only in certain basal cells (scored as 1), 12 (14%) podoplanin expression extending to suprabasal layer at one area (scored as 2), and 25 (30%) at two or more areas (scored as 3), as illustrated in Figure 1. According to the criteria previously described [13], lesions with scores ≥ 2 were considered as podoplanin-positive based on the notion that lesions with extensive podoplanin expression beyond the basal layer may reflect clonal expansion and are more prone to progression. Thus, 37 (44%) of the 84 lesions were classified as podoplanin-positive and the remaining 47 (56%) lesions were considered podoplanin-negative.

There was no statistically significant correlation between podoplanin status and the severity of dysplasia: 5 (36%) of the 14 lesions with mild dysplasia, 9 (35%) of the 26 lesions with moderate dysplasia, and 23 (52%) of the 44 lesions with severe dysplasia/carcinoma "in situ" exhibited positive podoplanin expression (*P *= 0.28).

The expression of podoplanin in the dysplastic lesions was correlated with the risk of progression to laryngeal cancer (Table 2). During the follow-up period, 33 of the 84 patients (39%) developed an invasive carcinoma at the same site of the previous premalignant lesion. The evolution to invasive carcinoma in relation to the histopathological diagnosis is shown in Table 2. In this cohort study, the group of patients with mild dysplasia showed the highest rate of progression to malignancy although the differences were not statistically significant. The mean time to cancer diagnosis in the cases that progressed was 28 months (range 11 to 66 months).

**Table 2:** Evolution of the premalignant lesions in relation to histopathological diagnosis and podoplanin expression

Characteristic	No. of cases	Progression to carcinoma (%)	*P*
Histopathological diagnosis			
- Mild dysplasia	14	7 (50)	0.27
- Moderate dysplasia	26	7 (27)	
- Severe dysplasia	44	19 (43)	
			
Podoplanin expression			
- Negative (scores 0-1)	47	14 (30)	0.071
- Positive (scores 2-3)	37	19 (51)	

**Figure 2:**
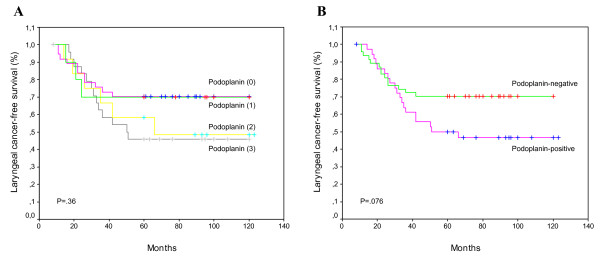
**Kaplan-Meier cancer-free survival curves categorized by podoplanin scores (A), and by podoplanin expression (positive *versus *negative) (B)**. *P *values were estimated using the log-rank test.

The risk for laryngeal cancer development in patients whose lesions scored 0 or 1 seems lower than in those whose lesions scored 2 or 3, especially after the first 3 years of follow-up (Fig. 2A). Consistent with these results, patients with podoplanin-positive lesions had a higher laryngeal cancer incidence than did those with podoplanin-negative lesions (Fig. 2B), although the differences did not reach statistical significance (HR = 1.84; 95%CI, 0.92-3.68; *P *= 0.076). At 5 years after the patients were diagnosed, 30% of the patients with negative podoplanin expression developed laryngeal cancer compared with 51% of the patients with positive podoplanin expression (*P *= 0.071; Table 2).

### Podoplanin expression in laryngeal squamous cell carcinomas

As expected podoplanin was highly expressed in lymphatic endothelial cells, whereas in histologically normal squamous epithelium adjacent to the tumours podoplanin expression was not detectable or extremely low in some basal cells. In laryngeal carcinomas, podoplanin expression was generally heterogeneous and exhibited two different patterns, similar to previous observations in oral cancer [12]: 20 (38%) cases showed diffuse expression in most tumour cells (Fig. 1E) and 33 (62%) cases showed focal expression at the proliferating periphery of the tumour cell nests with no expression in the central areas (Fig. 1F). In the latter cases the central areas often contained more differentiated cells, mimicking the pattern seen in the dysplastic epithelium. Two (4%) of the tumours showed no podoplanin expression, 31 (58%) had weak or moderate expression (IRS scores 1-6), and 20 (38%) had high expression (scores 7-15). For statistical purposes, tumours with scores equal or lower than 6 (median value) were considered to have low podoplanin expression, whereas those with scores higher than 6 were considered high expression.

The relationships between podoplanin expression and the clinico-pathological variables are shown in Table 1. Higher levels of podoplanin expression were observed in glottic carcinomas (*P *= 0.01). Podoplanin expression significantly decreased as T classification increased (*P *= 0.033) and in consequence there was also a significant inverse association of podoplanin expression with disease stage (*P *= 0.006), with most stage IV tumours showing low podoplanin expression. Moreover, well-differentiated carcinomas exhibited significantly higher levels of podoplanin, compared to moderately or poorly differentiated carcinomas (*P *= 0.04). No correlation between podoplanin expression and nodal metastasis was observed (*P *= 0.53).

**Figure 3:**
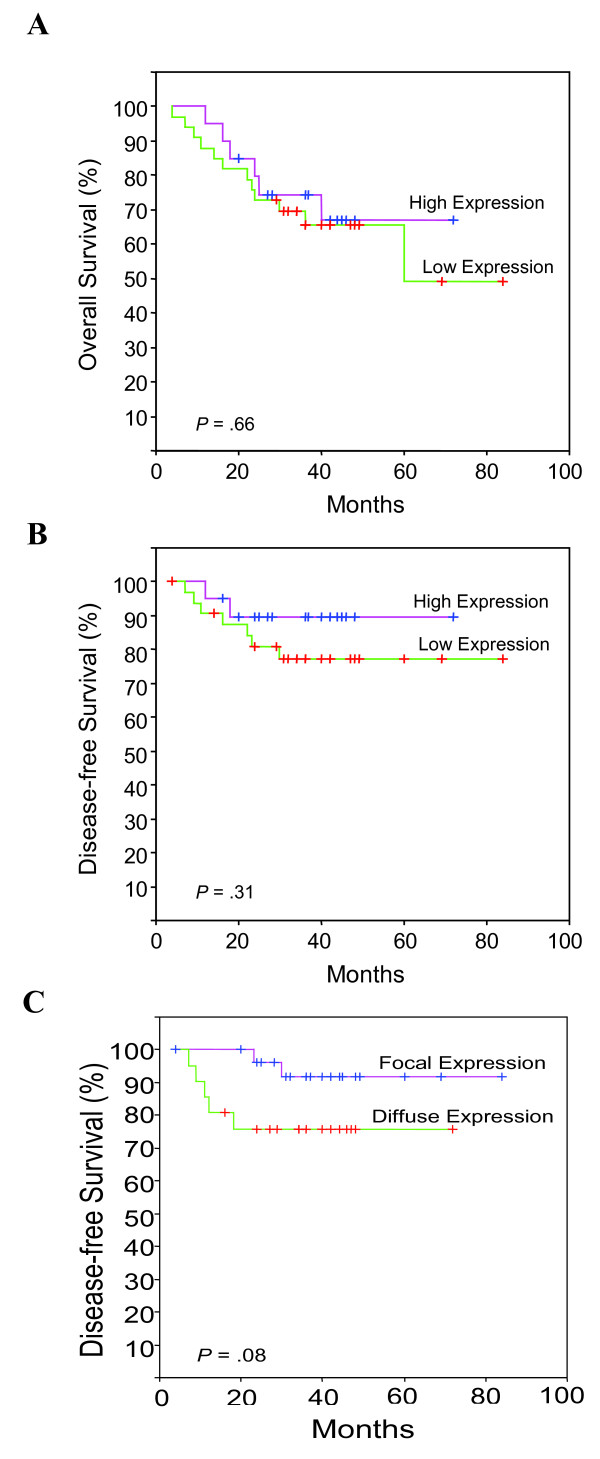
**Kaplan-Meier overall (A) and disease-free survival (B) curves categorized by podoplanin expression (high *versus *low) and disease-free survival (C) by podoplanin expression pattern (focal *versus *diffuse)**. *P *values were estimated using the log-rank test.

Patients were followed-up for a minimum of 36 months. The median follow-up of the whole series was 36 months (range, 4-84 months), and the median follow-up of the patients alive at the last visit was 43 months (range, 36-84 months). During this follow-up period, seven (13%) cases developed loco-regional recurrence, and 4 (8%) cases distant metastasis. Podoplanin expression was not associated with tumour recurrence (*P *= 0.42, Table 1) and no significant differences in overall survival were observed when comparing patients with high *versus *low podoplanin expression (Log-rank test, *P *= 0.66; Fig 3A). Nevertheless, patients with low levels of podoplanin had a poorer disease-specific survival, although this difference did not reach significance (Log-rank Test, *P *= 0.31; Fig. 3B). Furthermore, those cases showing diffuse podoplanin staining had a poorer disease specific-survival than the cases that displayed a focal expression pattern in the periphery of tumour nests, but there was only a borderline statistical difference (Log-rank test, *P *= 0.08; Fig 3C).

Multivariate analysis using the Cox proportional hazards model included tumour site, T classification, nodal metastasis, stage, and degree of differentiation and podoplanin expression. This model showed that only the presence of cervical lymph node metastasis was a significant independent predictor of reduced disease-specific survival (HR = 13; 95%CI, 2.7-63; *P *= 0.0014) or overall survival (HR = 6; 95%CI, 2.3-15.6; *P *= 0.0002).

## Discussion

The physiological function of podoplanin is still unknown. Podoplanin-deficient mice die at birth owing to respiratory failure exhibiting a phenotype of dilated, malfunctioning lymphatic vessels and lymphoedema [16]. In addition, podoplanin can induce platelet aggregation *in vitro *[17]. In the pathological situations studied thus far, the mouse homologue of podoplanin (PA2.26, OTS-8) is induced in mouse skin during tissue regeneration after wounding and treatment with carcinogenic phorbol 12-myristate 13-acetate [18]. OTS-8 is also induced by 12-Otetradecanoylphorbol- 13-acetate in mouse osteoblastic cells and is constitutively expressed in oncogenic Ras-transformed cells [19]. Podoplanin has also been found to promote tumour cell invasion by inducing collective cell migration via the down-regulation of the activities of small Rho family GTPases [20]. These findings suggest a role of podoplanin in tissue development and repair as well as in carcinogenesis and malignant progression.

In this study we found that podoplanin is expressed in a high percentage of dysplastic lesions and squamous cell carcinomas of the larynx. This is not surprising since podoplanin expression is mainly detected in squamous cell cancers, CNS tumours and germinal neoplasia; in contrast, expression of podoplanin has not been found in the majority of adenocarcinomas, including lung, colon and prostate cancers [20]. Podoplanin expression has been previously explored in other head and neck squamous cell carcinomas, such as oral and hypopharyngeal carcinomas [13], as well as in oral premalignancies [14]. However, our study is the first to analyse podoplanin expression in both laryngeal premalignant lesions and laryngeal carcinomas and its relationship with clinico-pathological parameters and prognosis.

In oral leukoplakia, high podoplanin expression has been associated with an increased risk of progression to invasive cancer, suggesting that podoplanin could serve as a powerful biomarker to predict the risk for oral cancer development in patients with oral leukoplakia [14]. This evidence supports the importance of podoplanin in oral tumourigenesis and malignant transformation. Similarly, our findings indicate that podoplanin is also frequently abnormally expressed in the early stages of laryngeal tumourigenesis and patients carrying podoplanin-positive dysplastic lesions (scores 2-3) exhibit a higher incidence of laryngeal cancer than patients with negative expression (51% *versus *30%), although these differences did not reach statistical significance (*P *= 0.071). Since podoplanin expression was not associated with the severity of dysplasia, this trend towards a poorer prognosis for patients with podoplanin-positive dysplasias suggests a role for podoplanin in the progression to laryngeal cancer. In this study cohort, the histology of the lesions does not have a significant role in assessing laryngeal cancer risk, and podoplanin seems a stronger predictor. The unexpected high proportion of mild dysplasias with progression to laryngeal carcinoma in our cohort could be due to pure chance, given the limited number of patients with this diagnosis. However, this also underscores the limited value of histopathological classification in predicting outcome. These data suggest the utility of podoplanin as a biomarker for cancer risk assessment providing additional value beyond current clinical and histopathological evaluations.

Nevertheless, podoplanin expression alone may not be sufficient to promote tumourigenesis because many of the lesions (44%) exhibit protein expression only in the basal layer cells, and this was independent of the severity of the dysplasia. Other factors are therefore needed to promote clonal expansion of the abnormal cells. The upward clonal expansion of the podoplanin-expressing cells in the epithelial layers supports this notion. Indeed, lesions with such clonal expansion carry a higher risk of laryngeal cancer development. The ability to detect the cells expanding beyond basal layers may allow us to visualise potential clonal expansion, possibly from stem-cell clones, during tumourigenesis [14]. In fact, podoplanin has been identified as a marker of tumour-initiating cells (TICs) in squamous cell carcinomas [21]. Tumourigenicity and capability of recapitulating human SCC are by definition properties of TICs. Premalignant lesions with podoplanin expression beyond the basal cell layer may represent truly early neoplastic lesions, enriched in TICs and with a higher risk of progression to invasive cancer.

In relation to the possible role of podoplanin in tumour progression, it has been reported that high podoplanin expression significantly associates with nodal metastasis and reduced survival in oral squamous cell carcinomas [13]. In marked contrast to these findings, we did not find a significant association of podoplanin expression with lymph node metastasis nor poor prognosis in laryngeal cancer. On the contrary, patients with high podoplanin expression showed a better disease-specific survival. This could be explained by the fact that podoplanin expression was higher in early-stage tumours and, interestingly, all cases that developed distant metastasis showed low podoplanin expression. Indeed, we found a significant inverse association of podoplanin expression with disease stage and T classification. Podoplanin expression was also significantly more frequent in glottic tumours, which are generally smaller and detected at an earlier stage than supraglottic tumours. Our results showing that podoplanin expression levels decreased with primary tumour size evidence a higher proportion of podoplanin-positive cells in small tumours and podoplanin-positive staining in those tumours could reflect a higher proportion of TICs, rather than a higher invasive potential. In addition, podoplanin expression was also significantly higher in well-differentiated tumours, which are usually less invasive than those poorly differentiated. We also observed a trend towards better disease-specific survival for patients with a focal expression of podoplanin in the periphery of tumour nests, defined in some works as the invasive edge of the tumours [20]. There are contradictory data regarding the clinical significance and biological role of podoplanin expression in squamous cancers, with contrasting results depending on the tumour sites studied. Thus, in squamous cell carcinoma of the uterine cervix, low levels of podoplanin were significantly associated with the presence of lymphatic invasion and lymph node metastasis, as well as with shorter survival and higher risk of tumour recurrence [22,23]. In addition, tumour emboli within lymphatic spaces as well as metastatic cells from lymph nodes showed no podoplanin immunostaining in the vast majority of tumours, even in those cases with positive expression in the main tumour mass [23]. In squamous cell carcinomas of the lung [24], patients who had podoplanin-positive tumours, and especially those with focal expression in the periphery of tumour nests (named hierarchical distribution pattern by the authors) showed a significantly better overall survival than those with podoplanin-negative tumours. In addition, podoplanin expression in these tumours inversely correlated with lymphatic invasion and lymph node metastasis. This is in marked contrast to the observations made by Yuan *et al *[13] in oral squamous cell carcinomas, although in good agreement with the results presented herein. The results presented by Shimada *et al *[24]and our results suggest that squamous cell carcinomas with focal expression of podoplanin in the periphery of tumour nests (which is equivalent to the hierarchical distribution pattern) may indicate lower biological aggressiveness. According to this, it is plausible that squamous cell carcinomas showing focal expression pattern represent a well-organised tumour group based on the TICs concept, whereas squamous cell carcinomas with a diffuse expression pattern could reflect disordered tumours in terms of the developmental hierarchy.

Hence the role of podoplanin in tumour initiation and progression remains elusive. Its involvement in tumour metastasis, however, has been demonstrated in an experimental model to be due to its platelet aggregation-inducing activity leading to pulmonary retention of CHO cells that overexpress podoplanin [25]. It has also been demonstrated that podoplanin contributes to tumour invasion by binding ERM proteins to activate RhoA resulting in epithelial-mesenchymal transition [26]. Although podoplanin-positive TICs in squamous cell carcinomas may use these mechanisms to initiate and sustain tumour growth, they may also proliferate rapidly through the activation of the SHH signalling pathway [21]. In addition to these intrinsic mechanisms, the microenvironment also influences the ability of TICs to generate tumours [21]. Considering their localization, it has been proposed that TICs may be regulated by stromal cells, which is comparable to the regulation of stem cells by their environmental niche [21]. Histologically, podoplanin-positive cells were specifically located in most cases at the basal region of squamous cell carcinoma tumour nests, close to the surrounding stromal cells and the tumour-microenvironment interaction plays a decisive role in cancer progression. It has been recently described that positive podoplanin expression in stromal fibroblasts exerts a protective role against cell invasion and is a significant indicator of good prognosis in patients with advanced colorectal cancer [27].

## Conclusions

We have demonstrated for the first time that podoplanin is expressed in a high percentage of laryngeal dysplasias and laryngeal squamous cell carcinomas. Podoplanin-positive dysplasias had a higher risk of progression to invasive carcinoma than those with negative expression, although the differences did not reach statistical significance. Prospective studies involving larger numbers of patients are needed to further evaluate the clinical utility of podoplanin as a biomarker for laryngeal cancer risk assessment providing additional value beyond the clinical and histological markers. Podoplanin expression in laryngeal squamous cell carcinomas, however, diminishes during tumour progression and does not correlate with invasive potential. Taken together, these data support a role for podoplanin expression in the initiation rather than in the progression of laryngeal cancers.

## Declaration of Competing interests

The authors declare that they have no competing interests.

## Authors' contributions

JPR conceived of the study, and participated in its design and coordination, in the quantification of the immunostainings and drafted the manuscript. DG participated in the quantification of the immunostainings and in the statistical analysis. MVG participated in the design of the study and in the acquisition of the clinical and pathological data. GM participated in the acquisition of the clinical data and in the immunohistochemical staining. MFF participated in the histological analysis of the samples and in the quantification of immunostainings. JGP participated in the design of the study, in the statistical analysis and helped to draft the manuscript. All authors read and approved the final manuscript.
